# Neuropsychological Assessment and Rehabilitation of Patients with Impaired Monitoring as a Component of Executive Functions

**DOI:** 10.11621/pir.2026.0107

**Published:** 2026-03-15

**Authors:** Ivan A. Larin, Alina Yu. Komarova, Yulia A. Sandalova, Maria S. Kovyazina, Nataliya A. Varako, Jill Winegardner, Vadim D. Daminov

**Affiliations:** a *Lomonosov Moscow State University, Russia*; b *Russian Center of Neurology and Neurosciences, Moscow, Russia*; c *Federal Scientific Center for Psychological and Interdisciplinary Research, Moscow, Russia*; d *University Hospitals Cleveland Medical Center, USA*; e N.I. Pirogov National Medical and Surgical Center, Moscow, Russia

**Keywords:** executive functions, monitoring, neuropsychological rehabilitation, P.YaGalperin’s theory, restorative learning

## Abstract

**Background:**

There is a high prevalence of impairments in executive functions in various neurological diseases (stroke, traumatic brain injury, neurodegenerative disorders), which severely limits patients’ independence and quality of life. The study integrates the theory of the systematic, step-by-step formation of mental actions and concepts by Galperin, Luria’s approach to neuropsychology, and modern foreign rehabilitation techniques (*e.g.*, strategies from the “Goal Management Training” program).

**Objective:**

To assess the impact of a specialized neuropsychological training program on monitoring process indicators in patients with brain damage of various etiology.

**Design:**

The study involved 48 patients with brain damage of various etiology, aged from 23 to 74 years (*M*_age_ = 57.6; *SD =* 11.13; 17 women and 31 men). The patients were divided into an experimental group and a control group.

**Results:**

A specialized training program aimed at improving monitoring process indicators in patients with impaired executive functions was developed, as well as a neuropsychological diagnostic protocol for assessing monitoring. We did not observe a training effect when comparing the monitoring index values before and after the training within the experimental group. However, we found significant differences between the experimental and control groups when analyzing the indices based on the results of the post-test assessment (U = 170, *p =* .008, *p*_bonferroni_ = .032), along with significant improvements in five monitoring indicators and the total Frontal Assessment Battery score in the patients of the experimental group.

**Conclusion:**

The training effect, when comparing the experimental and control groups at the post-test, may be interpreted within the framework of intra-systemic semantic reorganization and the initial formation of a new mental action of selection and implementation of a solution in which monitoring actively participates. This interpretation is preliminary and requires further empirical validation. In future research, we plan to focus the training on the formation of the mental action of control.

## Introduction

Executive functions serve regulatory, modifying, and directive roles for other mental and non-mental processes (Andrewes, 2015). Contemporary neuropsychological theories primarily distinguish components of executive functions (Andrewes, 2015; Diamond, 2013; Miyake et al., 2000; Stuss, 2011). A.R. Luria (2009) considered executive functions within the third functional brain unit. These functions are associated with the neuropsychological factor of “programming and control” attributed to the prefrontal (convexital) areas of the cerebral hemispheres (Khomskaia, 2006). Deficits in executive function are noted not only following lesions in the frontal areas, but also in other brain regions (*e.g.*, the parietal lobes, thalamus, or striatum) (Tyburski et al., 2018). Impairments of these functions are prevalent in neurological disorders, including stroke, traumatic brain injury, Huntington’s disease, Parkinson’s disease, Alzheimer’s disease, and frontotemporal dementia (Andrewes, 2015).

Among executive functions, the monitoring of performed activities is highlighted (Diamond, 2013; Khomskaia, 2006; Korsakova, 2012; Luria, 2009; Miyake et al., 2000; Stuss, 2011). For instance, Luria (2009) identified it as one of the core tasks of the third functional brain unit (programming, regulation, and control), while Stuss and Alexander (2007) discussed monitoring as a component of executive functions, localized in the right lateral prefrontal cortex, responsible for checking task performance over time for “quality control” and behavioral adjustment.

In rehabilitating patients with impaired executive functions, emphasis is placed on replacing internal regulation of mental processes with external support and presenting action programs in an externalized, detailed format (Tsvetkova, 2004). A primary method for restoring these functions involves creating conditions where patients can internalize action plans initially managed solely by the psychologist (Baulina et al., 2023). Within the Soviet and Russian neuropsychological tradition, the restoration of executive functions is primarily considered within the framework of restorative learning (Tsvetkova, 2004), including the application of Galperin’s method of the systematic, stage-by-stage formation of mental actions. International approaches note numerous methods for rehabilitating such patients with various training programs (Kennedy et al., 2008). According to the meta-analysis by Cicerone and colleagues (2011) of rehabilitation studies on patients with traumatic brain injury, only metacognitive strategy training merits a “practice standard” recommendation. A similar conclusion was reached in the meta-analysis by Kennedy et al. (2008), emphasizing that metacognitive strategy training should be used with adults up to middle age who have sustained a traumatic brain injury, helping them in overcoming difficulties related to problem-solving, planning, and organization. Notably, their meta-analysis showed that metacognitive strategy training, compared to control interventions, significantly improved activity and participation outcomes but not impairment-level measures, highlighting the need to examine specific components such as monitoring in detail.

Another program used in rehabilitating patients with executive function deficits is “Goal Management Training” (GMT). It has been suggested that GMT is more likely to be successful when combined with other strategies, *e.g.*, problem-solving therapy, and when practiced in real-life situations (Krasny-Pacini et al., 2014). Developed by Levine and colleagues (2000), GMT targets components of executive functions like planning, decision-making, goal attainment, and problem-solving. A version of this program, offered by Winson et al. (2017), involves six stages: defining the main goal, generating potential solutions, weighing the pros and cons of each option, selecting a solution and planning steps, implementation with monitoring, and outcome evaluation. Winson et al. refer to the conceptual framework of Stuss, outlining specific strategies targeting distinct domains of executive functions. Based on these strategies, Shcherbakov et al. (2021) developed a neuropsychological rehabilitation training incorporating “Stop/Think” and “Zoom In/Out” techniques, along with a modified procedure derived from Goal Management Training.

A promising direction is the integration of modern rehabilitation methods with the fundamental principles of Galperin’s theory. For example, Kovyazina et al. (2025) designed a rehabilitation training program for patients with neglect syndrome that incorporates techniques by Winson et al. (2017) within the framework of the stage-by-stage formation of mental actions. The present study continues this line of research. Our training program is based on the principles of restorative learning (Luria, 1948; Tsvetkova, 2004), the method of step-by-step formation of mental actions and concepts by Galperin (Galperin, & Kabyl’nitskaia, 1974; Talyzina, 1998), and rehabilitation strategies developed by Winson and colleagues (2017).

## Methods

### Participants

The sample consisted of 48 patients (17 women and 31 men), aged between 23 and 74 years, with neurological disorders of various etiology. The mean age was 57.6 years (*SD =* 11.13). The experimental group had a mean age of 56.7 years (*SD =* 11.66), and the control group had a mean age of 58.6 years (*SD =* 10.69). Regarding clinical characteristics, in the experimental group (*n =* 25), diagnoses were: ischemic stroke (15; 60%), traumatic brain injury (TBI) (5; 20%), hemorrhagic stroke and subarachnoid hemorrhage (SAH) (4; 16%), and mixed ischemic-hemorrhagic (1; 4%). In the control group (*n =* 23): ischemic stroke (18; 78.3%), hemorrhagic stroke and SAH (3; 13%), post-TBI sequelae (1; 4.3%), and cerebral aneurysm (1; 4.3%)

Data on the exact time elapsed between the brain lesion event and the baseline assessment were not systematically recorded, although all patients were in the chronic stage of recovery (at least three months post-event), and this limitation should be considered when interpreting the findings.

The patients were divided into two groups: an experimental group (*n* = 25) and a control group (*n* = 23). Participants were assigned to either the experimental or control group based on the order of admission to the rehabilitation center and the availability of training slots. This resulted in a quasi-experimental design, as randomization was not feasible due to the clinical setting and the need to ensure that all eligible patients could receive the intervention if they wished. Importantly, the groups were comparable at baseline in terms of age, sex, and diagnosis distribution, reducing the likelihood of systematic bias.

The inclusion criteria for both groups were mild to moderate impairments in executive functions, right-handedness, and a clear state of consciousness. Exclusion criteria were as follows:

1. Impairments in impressive or expressive oral speech, or severe reading disorders;
2. Severe neurodynamic and modality-nonspecific memory impairments;
3. Severe modality-specific impairments in auditory-verbal memory;
4. Perceptual disorders.

### Procedure

The research was carried out from January 2024 to April 2025 at the Department of Medical Rehabilitation of Patients with Impaired Central Nervous System function, of the Pirogov National Medical and Surgical Center of the Ministry of Health of the Russian Federation.

We conducted an intergroup field experiment. The independent variable was the application of an intervention in the form of the executive functions training program. The dependent variable was the change in quantitative scores on the diagnostic measures of executive functions between pre- and post-assessments.

The experimental group underwent the executive functions training, and the control group received no experimental intervention. The experimental design can be classified as a pretest-posttest control group design (Kornilova, 2022). The time interval between the pre- and post-assessment was approximately 2–3 weeks for all patients, corresponding to the standard duration of inpatient rehabilitation. Both groups were assessed using the same diagnostic protocol at admission (pretest) and before discharge (posttest).

#### Research Methods

A comprehensive assessment of executive functions was conducted using tests from the Frontal Assessment Battery (FAB), the Tower of London task (Culbertson, & Zillmer, 1999), the Color-Word Interference Test (CWIT) from the Delis-Kaplan Executive Function System (Delis, Kaplan, & Kramer, 2001), as well as tests of proverb interpretation from Luria’s classical neuropsychological assessment, the “Strange Stories” test (Happé, 1994), and “Bidstrup’s picture sequences” task.

This test battery was designed to provide a comprehensive assessment of executive functions, alongside measurements of thinking, memory, attention, and the motor and visuospatial skills. Tasks involving memorization of phrases and a graphomotor test were included.

We hypothesized that the most sensitive indicators of monitoring from the FAB would be words from other grammatical categories, words not beginning with a certain letter, repetitions, proper nouns from the “Lexical Fluency” indicator; echopraxia from the “Conflicting Instructions”; and echopraxia, systematic perseverations without error awareness from the “Go-No Go”. These errors reflect disrupted adherence to instructions and lack of error awareness. Patients were instructed to inform the examiner if they made a mistake during the task.

In the Tower of London test (Culbertson, & Zillmer, 1999), the indicator chosen to assess monitoring was the total number of rule violations in the main series. This number was divided by the number of attempted tasks, as some patients refused to complete certain items. For the Color-Word Interference Test (CWIT) from the Delis-Kaplan system, the number of uncorrected errors in the main condition was selected as the criterion for evaluating monitoring.

We also used the “Strange Stories” test – a translated version of the instrument developed by Happé (Happé, 1994). In the “Strange Stories” (“The Gift” and “The Thief ”), patients were required to read a text and then answer questions to assess their understanding of the characters’ behavior and emotions. For “Bidstrup’s picture sequences” (“In the Restaurant” and “The Snowman”), criteria similar to those for the “Strange Stories” were identified. To assess monitoring in these two tasks, indicators such as digressions into irrelevant associations and repetition of an incorrect answer after feedback were used, as they reflect a disruption in the ongoing verification of task performance for “quality control” and behavioral adjustment (Stuss & Alexander, 2007).

The indicators we identified for assessing monitoring in the FAB’s tasks, the tests of proverb interpretation, the “Strange Stories”, and “Bidstrup’s picture sequences” were rated on a scale from 0 to 2, where 0 corresponded to no impairment, 1 to moderate impairment or observed compensatory strategies, and 2 to severe deficits.

#### Methods of exposure

The specialized neuropsychological training program consisted of four group sessions (60 minutes each). The training sessions for the experimental group were conducted on weekdays after 14:00 as an additional activity. The afternoon timing was chosen to fit the hospital schedule; however, it may have contributed to fatigue, which could have affected participants’ engagement during the sessions. Each session focused on one of the four components of executive functions outlined by Cicerone and colleagues (2006). The program included rehabilitation strategies developed by Winson et al. (2017) as well as our own techniques. During the second and third sessions, we applied P.Ya. Galperin’s method of the systematic, stage-by-stage formation of mental actions and concepts (Galperin & Kabyl’nitskaia, 1974; Talyzina, 1998). This was used to form the mental action of selecting and implementing a solution and to practice our modified version of the “Goal Management Training” (GMT) framework. In addition, our training was designed to foster social interaction; each session included psychoeducational components aimed at enhancing patients’ awareness of their deficits.

As an illustration, we will describe the second and third sessions, which were designed to practice strategies for overcoming difficulties with monitoring and to form the mental action of selecting and implementing a solution.

The second session, focused on “thinking and planning”, began with a brief theoretical psychoeducation block. Throughout the session, we worked with the patients to develop a scheme to address their “thinking and planning” difficulties. We gradually incorporated three techniques into this scheme: “Stop and Think”, “Pros and Cons”, and “Step by Step”. The scheme development was based on a modification of GMT presented by the Oliver Zangwill Centre (Winson et al., 2017).

The “Stop and Think” technique was practiced using a task involving packing a suitcase. The task instruction was: “You are going on a 10-day seaside trip and need to pack 10 essential items.” Patients were given cards depicting various objects. They were required to define their main goal using the “Stop and Think” technique and then select cards appropriate for that goal. The second technique, “Pros and Cons”, involved having patients evaluate the advantages and disadvantages of a potential decision before committing to it. To practice this, patients worked on a task requiring them to choose the optimal type of transport from Moscow to Saint Petersburg. The “Step by Step” technique highlighted the importance of decomposing a decision into concrete, sequential steps. To train this technique, patients completed a “metro” task, where they had to detail the steps to travel from one metro station to another, including a required transfer.

The third session was focused on further practicing the scheme and the three techniques (“Stop and Think”, “Pros and Cons”, and “Step by Step”) introduced in the second session. The goal of this session was to form a new mental action termed “selecting and implementing a solution”, which involved the process of monitoring. According to Galperin’s theory of the systematic, stage-by-stage formation of mental actions, the formation of a new mental action requires passing through five main stages (Galperin, 2023a). His later work emphasizes motivation as another crucial condition for the formation of mental actions (Galperin, 2023b).

The formation of the motivational basis for the action began with a group discussion. It is assumed that such a discussion, positive feedback from patients, and the opportunity to share one’s thoughts help foster motivation for the subsequent sessions (Grigor’eva, Kovyazina, & Tkhostov, 2013). Furthermore, patient psychoeducation served to establish this motivational foundation. At the beginning of the third session, we briefly reviewed the definition of the “thinking and planning” domain with the patients.

The second stage in forming a mental action involves creating an orienting basis for the action, which the patient will subsequently follow (Galperin, 2023a). During the rehabilitation sessions, we employed the orienting basis of an action of the third type, characterized by its completeness, generalizability, and the learner’s independence in applying it (Talyzina, 1998). This was realized through the scheme we developed with the patients in the second session. That scheme provided a complete set of operations necessary for performing the target action. The psychologist demonstrated how to apply the techniques using example tasks. The critical aspect of that stage was the externalization and fixation of the activity’s structure (Talyzina, 1998), which we achieved by using a presentation that sequentially displayed the steps of the scheme as the psychologist explained them.

The third stage was characterized by the formation of the action in its materialized form. In addition to the scheme, patients were given practical assignments. For example, during the third session, patients were provided with printed copies of the scheme and asked to cross out each step as they completed it. They worked on practical tasks such as planning a trip or a birthday party at a country house. We would first ask patients what issues needed to be resolved before the trip and what goals should be set. Questions formulated by the participants, such as “where to go”, were then defined as the main tasks for the scheme. To solve the main task, patients had to go through all the steps of the scheme. We asked them to verbalize each step before execution and then to cross it out or place a checkmark next to it upon completion. Subsequently, the action was detached from physical objects and transferred to the plane of external speech (Galperin, 2023a). Accordingly, in the latter half of the third session, patients no longer crossed out steps on the paper, but instead verbalized them before execution.

It was anticipated that by the end of the course, patients would be at the stage of external speech acts. However, they would not have fully completed the next stage of forming the action in external speech to oneself, nor the final stage of formation, where the action is carried out in the form of internal speech as an individual process requiring no external support (Talyzina, 1998).

## Results

Statistical analysis of the quantitative data was performed using Jamovi software. Reliability was assessed using Cronbach’s alpha and McDonald’s omega; non-parametric tests (the Wilcoxon T-test and the Mann-Whitney U-test) were employed for group comparisons.

The primary objective was to create a composite monitoring index. Although exploratory factor analysis (EFA) is often recommended for validating composite measures, its use requires an adequate sample size (minimum 5 observations per variable (Hair Jr et al., 1998)). With 10 indicators, at least 50 participants would be needed, yet our total sample was 48. Given the risk of unstable and overfitted results, we refrained from EFA and instead relied on theoretical grounding and internal consistency (Cronbach’s α, McDonald’s ω) to support the index.

To ensure its statistical reliability and internal consistency, Cronbach’s alpha coefficient was calculated. Prior to summing the scores from different tasks, the data were standardized, as the various parameters were measured on different scales. For instance, the number of uncorrected errors in the main condition of the “Color-Word Interference Test” was an interval-scale measure, whereas digressions into irrelevant associations in the “Bidstrup’s picture sequences” task were measured on an ordinal scale (0 to 2, with 0 indicating no impairment).

Thus, the final composite monitoring index included the following standardized scores from ten tasks: the score from the “Conflicting Instructions”; the total score from the “Go-No Go” task (both from the FAB); the repetition of incorrect answers and digressions into irrelevant associations from the “Proverb Interpretation”, “Strange Stories”, and “Bidstrup’s picture sequences” tasks; as well as the total number of rule violations in the main series divided by the number of attempted items in the “Tower of London”; and the number of uncorrected errors in the “Color-Word Interference Test”.

The calculation of Cronbach’s alpha for the standardized scores of all indicators intended to measure monitoring revealed that the “internal consistency” of the scales α = .587. The monitoring score from the “Lexical Fluency” of the Frontal Assessment Battery (FAB) correlated negatively with the total scale. Consequently, it was decided to exclude this indicator from the composite index. Following its exclusion, the “internal consistency” of the scales reached α = .634. An additional analysis using McDonald’s omega yielded ω = .661.

We planned to conduct a repeated-measures ANOVA for the monitoring index. As a preliminary step, Levene’s test was applied to assess the homogeneity of variances (see *Table 1*). The results indicated a violation of the homogeneity of variances for the initial measurement (F = 4.249, *p =* .045). That finding invalidated the use of ANOVA in that case and required the application of non-parametric statistical tests.

**Table 1:** Homogeneity of Variances Test (Levene’s) for the Monitoring Index Scores

	**F**	**df1**	**df2**	***p***
Monitoring index (pretest)	4.249	1	46	.045
Monitoring index (posttest)	.446	1	46	.507

We applied the non-parametric Mann-Whitney U-test. The non-parametric Wilcoxon T-test for related samples to both the experimental and control groups was also conducted. Due to the execution of four pairwise comparisons (two between-group and two within-group), the Bonferroni correction was applied to control for Type I error, setting the significance level at α = .0125.

The non-parametric Mann-Whitney U-test was used to analyze the differences in the monitoring index scores between the control and experimental groups at both the pre- and post-assessments (see *Table 2*).

**Table 2:** Between-Group Comparisons of the Composite Monitoring Index at Pre-and Posttest Using the Mann-Whitney U Tests

	**Mann-T-Whitney test**	***P***	***p*_bonferroni_**	**Effect Size**
Monitoring index (pretest)	284.0	.190	1.0	.012
Monitoring index (posttest)	170.0	.008	.032	.409

**Figure 1.**
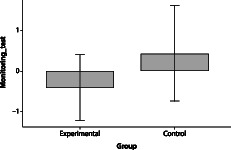
Boxplot of the composite monitoring index at pretest

**Figure 2.**
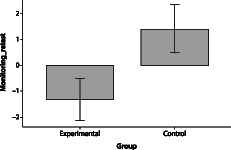
Boxplot of the composite monitoring index at posttest

The data indicated that the patients in the experimental and control groups did not differ significantly at the pre-assessment (U = 284, *p* > .05, *p*_bonferroni_ > .05). Descriptive statistics for the composite monitoring index, where higher values indicate poorer performance, are presented in *Table 3*, *Figure 1* and *Figure 2*. At pretest, the control group had a marginally higher mean score (*M* = .45, *SD* = 5.6) than the experimental group (*M* = −.41, *SD* = 4.05).

**Table 3:** Descriptive Statistics for the Monitoring Index

	**Group**	**Monitoring index (pretest)**	**Monitoring index (posttest)**
N	Experimental	25	25
	Control	23	23
Mean	Experimental	–.413	–1.306
	Control	.448	1.420
Median	Experimental	–1.34	–2.767
	Control	–.846	.19
Standard deviation	Experimental	4.054	4.163
	Control	5.604	4.597
Minimum	Experimental	–5.764	–5.548
	Control	–6.478	–5.548
Maximum	Experimental	9.699	9.003
	Control	11.526	10.201

At the post-assessment, however, the groups differed significantly (U = 170, *p =* .008, *p*_bonferroni_ = .032). The experimental group showed lower scores (*M =* −1.31, *SD =* 4.16) compared to the control group (*M =* 1.42, *SD =* 4.6). The effect size, calculated using the rank-biserial correlation coefficient, was .409, which corresponds to a moderate effect (Kerby, 2014).

At the post-assessment, however, the groups differed significantly (U = 170, *p =* .008, *p*_bonferroni_ = .032). The experimental group showed lower scores (*M =* −1.31, *SD =* 4.16) compared to the control group (*M =* 1.42, *SD =* 4.6). The effect size, calculated using the rank-biserial correlation coefficient, was .409, which corresponds to a moderate effect (Kerby, 2014).

Using the Wilcoxon T-test, we assessed whether there were changes in the monitoring index scores within the experimental and control groups between the pre- and post-assessments. No significant differences were found between the test and retest scores for the experimental group (W = 196, *p* >.05, *p*_bonferroni_ >.05) (see *Table 4*).

**Table 4:** Within-Group Comparisons of the Composite Monitoring Index (Pre- vs Posttest) Using the Wilcoxon Signed-Rank Test

	**Wilcoxon T-test**	***p***	**Pbonferroni**	**Effect Size**
Experimental group	196.0	.190	.76	.206
Control group	108.0	.820	1.0	–.217

In addition to the primary confirmatory analysis of the composite monitoring index, we conducted exploratory analyses of the individual monitoring indicators to identify potential patterns *(*see *Table 5*). Significant changes were found for five indicators, all of which showed strong effect sizes as estimated by the rank-biserial correlation coefficient.

We also used the Wilcoxon test to analyze changes in total FAB scores for both groups (*Table 6*). For the total FAB score, higher values correspond to better-preserved executive functions. The analysis revealed statistically significant differences for the experimental group (W = 196, *p* < .05) with a strong effect size (rank-biserial correlation, *r* = –.642).

**Table 5:** Exploratory analysis of Individual Monitoring Indicators: Within-Group Comparisons (Pre- vs Posttest) Using the Wilcoxon Signed-Rank Tests

	**Monitoring indicator**	**Wilcoxon T-test**	***P***	**Effect Size**
Experimental group	FAB: “Conflicting Instructions”: echopraxia	7.5	.212	.50
	FAB: “Go-No Go”: composite index	30.0	.63	– .091
	“Strange Stories”: error repetition	1.5	.681	0
	**“Strange Stories”: digressions into irrelevant associations**	**31.5**	**.021**	**.75**
	**“Bidstrup’s picture sequences”: error repetition**	**40.5**	**.012**	**.80**
	**“Bidstrup’s picture sequences”: digressions into irrelevant associations**	**72.5**	**.003**	**.859**
	“Comprehension of proverbs”: error repetition	17.500	.08	.667
	**“Comprehension of proverbs”: digressions into irrelevant associations**	**46.0**	**.028**	**.673**
	CWIT: uncorrected errors in the main series	170.5	.078	.348
	**“Tower of London”: total rule violations in the main series**	**164.0**	**.003**	**.726**

*Note. p-values less than .05 are highlighted in bold.*

**Table 6:** Within-Group Comparisons of Total FAB Scores (Pre- vs Posttest) Using the Wilcoxon Signed-Rank Test

	**Wilcoxon T-test**	***P***	**Effect Size**
Experimental group	196.0	.013	–.642
Control group	68.5	.521	.007

## Discussion

Cronbach’s α = .634 indicates acceptable internal consistency, although a larger sample would provide a more stable estimate. This is consistent with findings in other studies. For instance, when constructing indices for specific neuropsychological factors in child samples, some indices also demonstrated only acceptable values (*e.g.*, an index for serial organization of movements with α = .63) (Akhutina, 2022). However, in a more recent study by Bukinich et al. (2022) confirmatory factor analysis showed a good fit between the empirical data and the authors’ model of indicators and factors, i.e., various components of higher mental functions. Bukinich and colleagues conclude that the indices possess sufficiently high structural validity, even those for which the Cronbach’s alpha value had previously been only acceptable (Akhutina, 2022). The lexical fluency indicator was excluded from the composite monitoring index due to its negative correlations with other measures, consistent with the theoretical distinction between energization (assessed by verbal fluency) and monitoring (Stuss et al., 2006). This theoretical distinction may explain the negative correlations, as lexical fluency assesses energization— the capacity to generate and maintain actions or mental processes (Cicerone et al., 2006) — rather than monitoring, underscoring the need for future studies with separate measures of both components. After its exclusion, internal consistency reached an acceptable level (Cronbach’s α = .634), further supported by McDonald’s ω = .661.

The significant between-group difference in the monitoring index supports the presence of an intervention effect (see *Table 2*); the non-significant within-group change in the experimental group (see *Table 4*) may reflect insufficient power. To determine whether our sample size was adequate to detect a meaningful within-group effect, we conducted an a priori power analysis using G*Power. Based on the meta-analysis of metacognitive strategy interventions by Kennedy et al. (2008), which reported a pooled effect size of Hedges’ g = .41 for impairment-level outcomes, we estimated the sample size required for a paired Wilcoxon signed-rank test. With α = .05 and β = .80, the analysis indicated that a minimum of 40 participants would be necessary to detect an effect of this magnitude. Given that our experimental group comprised only 25 participants, the study may have been underpowered to detect within-group changes, which could account for the non-significant results.

We conducted a detailed exploratory analysis of each individual monitoring indicator within the experimental sample and found significant changes in specific parameters. These results indicate positive dynamics in the state of monitoring, yet improvements were confined to specific parameters.

The significant changes in the indicators of “repetition of an incorrect answer” and “digressions into irrelevant associations” are consistent with the hypothesis that patients were developing a new mental action of selecting and implementing a solution which was performed in an expanded form with verbalization by the end of the training. Specifically, the orienting basis of the action for this mental action included the step “stop and think”, where the patients were required to repeat the main task to themselves. For example, during the post-assessment tasks, such as “Bidstrup’s picture sequences”, some patients from the experimental group were observed whispering the task to themselves. The significant change in the “Tower of London” indicator may be related to the training’s emphasis on planning skills, which are central to both the GMT framework (Cicerone et al., 2006; Levine et al., 2000) and were specifically practiced during the formation of the mental action of selecting and implementing a solution. For example, the application of the “step by step” technique required patients to plan out their chosen solution in stages. However, as the specific mechanisms underlying this improvement were not directly assessed, this interpretation remains theoretical and requires further investigation.

We assessed changes in the total FAB score using the Wilcoxon T-test, which revealed significant differences in the experimental group, in contrast to the control group (see *Table 6*). We suggest that this indicates a positive influence of the training on executive functions as a whole.

In summary, the results based on the monitoring index from the comparison of experimental and control groups (see *Table 2*), individual tasks aimed at its assessment (see *Table 5*), and the total FAB score (see *Table 6*) point to positive dynamics in the state of monitoring and executive functions. These findings can be interpreted within the framework of intra-systemic reorganization (Luria, 1948; Tsvetkova, 2004). The strategies practiced during the sessions may have facilitated generalization of the material and contributed to a more conscious and voluntary level of performance, although this interpretation remains theoretical, as the mechanisms of reorganization were not directly assessed in the present study.

Furthermore, the goal of the second and third sessions was to form the new mental action named “selecting and implementing a solution”, which involved the process of monitoring. However, we cannot confirm that the patients progressed to the stage of mental action formation where the action is carried out in the form of internal speech as an individual process requiring no external support. Accordingly, by the end of the training, we had not succeeded in forming a mental action that was generalized, abbreviated, and automated. It may be hypothesized that, following the two-week training period, patients had reached the stage of external speech acts in the formation of the mental action. Whether participants internalized the action to the level of external speech, inner speech, or automated control remains an open question for future research.

Throughout the stage of external speech, the action of selecting and implementing a solution was performed by the patients in an expanded form, and its formation actively engaged monitoring as the process of checking task performance over time for “quality control” and behavioral adjustment. This suggests that the application of the method for the systematic, stage-by-stage formation of mental actions may have positively influenced the dynamics of monitoring.

For instance, during the post-test “Tower of London” task, one experimental patient verbalized the rules aloud and solved the task efficiently, whereas some controls needed repeated rule reminders. However, as the stage of mental action formation was not directly assessed, this interpretation remains tentative and requires further investigation.

The parameters of our training program are broadly consistent with those of studies included in recent meta-analyses of Goal Management Training (GMT) and metacognitive strategy interventions. Our sample of adults with stroke and TBI aligns with the populations most frequently studied in GMT research (Kennedy et al., 2008; Stamenova & Levine, 2019). Although our program was shorter (4 hours) than the 10–45 hours reported in meta-analyses, shorter GMT formats have also demonstrated significant results (Levine et al., 2000).

## Conclusion

The findings provide preliminary evidence for a training effect, as revealed by the comparison of monitoring index values between the experimental and control groups at the post-assessment. Furthermore, the exploratory analysis of differences in individual monitoring indicators within the experimental group indicated a trend toward change at the level of individual differences. The additional analysis also revealed that the total FAB score increased significantly in the experimental group, indicating an improvement in executive functions overall.

These results are consistent with the theoretical framework of intra-systemic semantic reorganization and may reflect the initial stages of forming the mental action of selecting and implementing a solution. However, as these constructs were not directly measured, this interpretation should be viewed as a hypothesis to be tested in future studies. As a result of the training, we cannot claim to have formed a mental action in its ideal, abbreviated, and automated form of control (Galperin, 1976). The mental action we aimed to form involved the monitoring process, but its object was not control per se; rather, it was the mental action associated with selecting and implementing a solution. Thus, this study has provided us with a better understanding of the future modifications required for a rehabilitation program to result in the formation of the mental action of control. By incorporating the principles of restorative learning more extensively, we plan to develop a specialized neuropsychological training program aimed at forming a mental action of control, where the object of the mental action would be control itself, and the goal would be the restoration of control as a parameter of executive functions.

Future study should also address the limitations of the present research through longitudinal designs with extended follow-up periods to assess the durability of training effects. The program should also be evaluated in other clinical populations (*e.g.*, Parkinson’s disease, multiple sclerosis, mild cognitive impairment) and should examine potential moderators of treatment response, such as baseline severity, lesion location, and level of insight.

## Limitations

The mental action of selecting and implementing a solution, developed during the four sessions, appeared to improve patients’ functioning, with observable benefits at the stage of external speech. Progressing through all six stages of Galperin’s method would likely require additional sessions, which is feasible within a two-week rehabilitation stay.

Several limitations should be acknowledged. First, the modest sample size limited statistical power to detect within-group changes. Second, factors such as fatigue, additional training load, time since injury, age, and gender were not fully controlled. Third, the absence of a follow-up assessment limits conclusions about long-term maintenance of the observed effects. Furthermore, potential practice effects cannot be ruled out, as alternate test forms were unavailable for most tasks; minor modifications to some tasks do not fully eliminate this concern. Finally, the lack of blinded evaluators introduces the risk of experimenter bias. These methodological issues should be addressed in future research.
